# Patterns of antibiotic administration in Chinese neonates: results from a multi-center, point prevalence survey

**DOI:** 10.1186/s12879-024-09077-7

**Published:** 2024-02-12

**Authors:** Jiaosheng Zhang, Li Lin, Gen Lu, Keye Wu, Daiyin Tian, Lanfang Tang, Xiang Ma, Yajuan Wang, Gang Liu, Yanqi Li, Jing Qian, Ping Wang, Qing Cao, Wenshuang Zhang, Lijuan Wu, Ligang Si, Yue Wu, Yuejie Zheng, Kunling Shen, Jikui Deng, Defa Li, Yonghong Yang

**Affiliations:** 1 Department of Infectious diseases, Shenzhen Children’s Hospital, Shenzhen, China; 2 Department of Respiratory, The Second Affiliated Hospital & Yuying Children’s Hospital of Wenzhou Medical University, Wenzhou, China; 3 Department of Respiratory, Guangzhou Women and Children’s Medical Center, Guangzhou, China; 4 School of Public Health and Emergency Management, Southern University of Science and Technology, Shenzhen, China; 5 Department of Respiratory, Children’s Hospital of Chongqing Medical University, Chongqing, China; 6 Department of Respiratory, Children’s Hospital Zhejiang University School of Medicine, Hangzhou, China; 7 Department of Respiratory, Children’s Hospital Affiliated to Shandong University & Jinan Children’s Hospital, Jinan, China; 8 Neonatal Department, Children’s Hospital Attached to the Capital Institute of Pediatrics, Beijing, China; 9 Department of Infectious Diseases, Beijing Children’s Hospital Affiliated to Capital Medical University, Beijing, China; 10 Department of Respiratory, Xi’an Children’s Hospital, Xi’an, China; 11 Department of Respiratory, Children’s Hospital Attached to the Capital Institute of Pediatrics, Beijing, China; 12 Neonatal Department, Beijing Obstetrics and Gynecology Hospital, Capital Medical University, Beijing, China; 13 Department of Infectious Diseases, Shanghai Children’s Medical Center, Shanghai Jiaotong University School of Medicine, Shanghai, China; 14 Department of Respiratory, Tianjin Children’s Hospital, Tianjin, China; 15 Clinical Laboratory, Bao’an Maternity & Child Health Hospital, Shenzhen, China; 16 Department of Pediatric, The sixth Hospital of Haerbin Medical University, Haerbin, China; 17 Department of Pharmacy, Shenzhen Children’s Hospital, Shenzhen, China; 18 Department of Respiratory, Shenzhen Children’s Hospital, Shenzhen, China; 19 Department of Internal Medicine, Shenzhen Children’s Hospital, Shenzhen, China; 20 Department of Respiratory, Beijing Children’s Hospital Affiliated to Capital Medical University, Beijing, China; 21 Department of Infectious Diseases, Shenzhen Children’s Hospital, Shenzhen, China; 22 Shenzhen Clinical College of Pediatrics, Shantou University Medical College, Shenzhen, China; 23 Clinical laboratory, Shenzhen Children’s Hospital, Shenzhen, China; 24 Beijing Pediatric Research Institute, Beijing Children’s Hospital Affiliated to Capital Medical University, Beijing, China

**Keywords:** Antibiotic, China, Neonates, Prescription, Pattern

## Abstract

**Objectives:**

In this study, we describe the patterns of antibiotic prescription for neonates based on World Health Organization’s (WHO) Essential Medicines List Access, Watch, and Reserve (AWaRe), and the Management of Antibiotic Classification (MAC) Guidelines in China.

**Methods:**

One-day point-prevalence surveys (PPS) on antimicrobial prescriptions were conducted on behalf of hospitalized neonates in China from September 1 and November 30, annually from 2017 to 2019.

**Results:**

Data was collected for a total of 2674 neonatal patients from 15 hospitals in 9 provinces across China of which 1520 were newborns who received at least one antibiotic agent. A total of 1943 antibiotic prescriptions were included in the analysis. The most commonly prescribed antibiotic was meropenem (11.8%). The most common reason for prescribing antibiotic to neonates was pneumonia (44.2%). There were 419 (21.6%), 1343 (69.1%) and 6 (0.3%) antibiotic prescriptions in the Access, Watch and Reserve groups, respectively. According to MAC Guidelines in China, there were 1090 (56.1%) antibiotic agents in the Restricted and 414 (21.3%) in the Special group.

**Conclusion:**

Broad-spectrum antibiotics included in the Watch and Special groups were likely to be overused in Chinese neonates.

**Supplementary Information:**

The online version contains supplementary material available at 10.1186/s12879-024-09077-7.

## Introduction

Widespread use of antibiotics, particularly broad-spectrum antibiotics, has contributed to an increase in antimicrobial resistance. With the unnecessary use of antibiotics, resistant bacteria become more abundant, and the resistome (the compendium of antibiotic resistance genes) within the gut microbiota has become enriched. Resistant bacteria in the gut are a source of resistant bacterial infections [1–3]. In addition to inducing antimicrobial resistance in children, antibiotics are closely associated with asthma, obesity, and other allergies [4–6].

In 2011, China launched a nationwide campaign to promote the sensible use of antibiotics and reduce antimicrobial resistance [7]. General antibiotic utilization rate and density has since decreased. However, antibiotic resistance has not yet been completely controlled. The number of multidrug- and carbapenem-resistant organisms is increasing annually [8]. In addition to antibiotic usage, the continued increase in the drug resistance rate may also be due to the application of broad-spectrum antimicrobial drugs.

Analysis of antibiotic prescriptions and indications targeting neonates is necessary for antimicrobial stewardship. There are limited published data on the general practice concerning indications for antibiotic prescriptions and antibiotic types prescribed for Chinese neonates. This data is the basis of information required for antimicrobial stewardship. Moreover, previous reports have focused on the risks of antibiotic prescription for neonates without the description of antibiotic classes, indications, etc.

In 2017, antimicrobial agents for children with the most frequent and severe bacterial infections were classified into three groups: Access, Watch and Reserve (WHO AWaRe) [9, 10]. These were developed by the WHO Expert Committee on the Selection and Use of Essential Medicines based on potential antimicrobial resistance, economy, and other factors. The Essential Medicines List (EML) was revised in 2019 and 2021. The Access group includes antibiotics that are readily available, affordable, and recommended as first-line treatment for common infectious diseases. In the Watch group, antibiotics with higher resistance potential were recommended only as first- or second-line treatments for a limited number of indications. The Reserve group includes antibiotics that should only be used as a last resort when other alternatives are inadequate or have already failed.

According to factors such as safety, efficacy, bacterial resistance, and price based on the Management of Antibiotic Classification (MAC) in China, antibacterial drugs are divided into the following three groups: Unrestricted, Restricted, and Special group [11]. The Unrestricted group included antibiotics, such as amoxicillin and ampicillin, which are safe, affordable, and effective, with little impact on bacterial resistance. The Restricted group included antibiotics with a higher potential for bacterial resistance and/or a higher price, such as ceftizoxime and ceftazidime. This Special group includes antibiotics, such as vancomycin and meropenem, which have serious adverse effects, are expensive, and/or have a high probability of inducing bacterial resistance.

This is a study including the data from a multi-center Point Prevalence Survey (PPS) on antibiotic administration to neonates in China. We aimed to present the patterns of antibiotic prescriptions for hospitalized Chinese neonates from 2017 to 2019 based on the class of the antibiotic agents, the WHO AWaRe classification and Management of Antibiotic Classification (MAC) in China.

## Methods

Three one-day point-prevalence surveys (PPSs) were conducted in September–November annually in 15 hospitals in China from 2017 to 2019. The participating hospitals chose any day within the 4-month survey period as the survey day to upload the antibiotic prescribing data.

Neonates and children (age range: birth to 18 years) who were hospitalized in participating hospitals were enrolled in the surveys. A standardized, fully structured, case-report form was designed for this survey. Web-based Electronic Data Capture (https://garpec-31.mobilemd.cn/login.aspx?relogin=true) was used to collect the respondents’ data, and pediatricians or members of each participating medical institution logged into the database and uploaded the prescription, indicators, antimicrobial and diagnosis data. Patient-specific demographic data, including age, body weight were collected by reviewing electronic medical records. Data were collected from hospitalized patients admitted to the internal medicine, surgery, and intensive care units (ICUs).

Only antibiotic prescriptions for neonates (age range: birth to 28 days) hospitalized in joined centers were analyzed in this study; data from children (age range 29 days to 18 years) were excluded.

Intravenous, intramuscular, and oral antibiotics were analyzed in this study, whereas inhaled or topically applied antibiotic prescriptions were excluded. Antifungal, antiviral, antitubercular agents (isoniazid, pyrazinamide, and ethambutol), and antiparasitic drugs were also excluded. Rifampicin was included in the database except if it was prescribed in combination with isoniazid, pyrazinamide, and ethambutol, in which case it was considered as anti-tuberculosis chemotherapy.

The detailed breakdown of antibiotics used in each group, based on the WHO AWaRe groups and China’s administrative categories of antibiotics, are shown in Supplementary Table 1.

Antibiotic use in neonates was expressed using drug utilization (DU) 90, a defined measurement where antibiotic agent that accounts for 90% of the total antibiotic prescriptions.

## Results

A total of 2674 patients from 15 hospitals in nine provinces across China participated in this survey. Of which 1520 newborns received at least one antibiotic agent on PPS days. The average antibiotic prescription rate was 56.8%, varying from 21.9 to 86.3% across hospitals. A total of 1943 antibiotic prescriptions were included in the analysis. The characteristics of antibiotic use in different hospitals are described in Table 1.

**Table 1:** The characteristics of participating hospitals and antibiotic prescribing for neonates from 2017 to 2019

Hospital code	Level	Province	Region	District	Total patients	Patients prescribed antibiotics	Rates of antibiotic therapy (%)	No. of antibiotic prescriptions	No. of antibiotic prescriptions per patient
H1	Tertiary	Chongqing	Southwest	Provincial	378	272	72.0	372	1.4
H2	Tertiary	Zhejiang	East	Provincial	523	282	53.9	336	1.2
H3	Tertiary	Shandong	East	Provincial	358	247	69.0	307	1.2
H4	Tertiary	Zhejiang	East	Municipal	246	123	50.0	177	1.4
H5	Tertiary	Beijing	North	National	104	88	84.6	121	1.4
H6	Tertiary	Guangdong	South	Municipal	249	94	37.8	112	1.2
H7	Tertiary	Shaanxi	Northwest	Provincial	146	102	69.9	111	1.1
H8	Tertiary	Beijing	North	Provincial	97	67	69.1	79	1.2
H9	Tertiary	Beijing	North	Provincial	54	42	77.8	71	1.7
H10	Tertiary	Shanghai	East	National	82	47	57.3	70	1.5
H11	Tertiary	Guangdong	South	Provincial	166	49	29.5	55	1.1
H12	Tertiary	Tianjin	North	Provincial	44	38	86.4	48	1.3
H13	Tertiary	Guangdong	South	District	119	26	21.8	36	1.4
H14	Tertiary	Heilongjiang	Northeast	Provincial	73	27	37.0	32	1.2
H15	Tertiary	Shandong	East	Provincial	35	16	45.7	16	1.0
					2674	1520	56.8	1943	1.3

## Reasons for antibiotics in Chinese neonates

The five most common reasons for the prescription of antibiotic for neonates were pneumonia (44.2%, 858/1943), sepsis (14.2%, 275/1943), prophylaxis for newborn risk factors (8.0%, 156/1943), gastrointestinal (GI) tract infections (5.8%, 112/1943), and surgical treatment (4.7%, 92/1943). The detailed reasons for antibiotics in Chinese neonates are shown in Table 2.

**Table 2:** Indications for antibiotics prescribing for Chinese neonates

Indications for antibiotics prescribing	Frequency	Percentage%
Pneumonia	858	44.2
Sepsis	275	14.2
Newborn Prophylaxis for Newborn Risk factors	156	8.0
Gastrointestinal tract infections	112	5.8
Treatment for surgical disease	92	4.7
Central nervous system infections	90	4.6
Bronchitis	79	4.1
Bloodstream infection	75	3.9
Pyrexia of Unknown Origin (PUO)	48	2.5
Medical prophylaxis	44	2.3
Skin/soft-tissue infections	34	1.7
Surgical prophylaxis	21	1.1
Urinary Tract Infections (UTI)	12	0.6
Newborn prophylaxis for maternal risk factors	12	0.6
Upper respiratory tract infections	9	0.5
Intrauterine infection	6	0.3
Unknown	6	0.3
Other*	5	0.3
Enhancing gastrointestinal tract motility	4	0.2
Joint/Bone Infections	2	0.1
Febrile neutropenia	2	0.1
Peritonitis	1	0.1
Total	1943	100

*‘Other’ refers to reasons for antibiotics that are not in the list designed for the survey

### Antibiotics prescribed for Chinese neonates

In total, 46 types of antibiotic agents were used, and 17 (37.0%) antibiotic agents accounted for 90% of antibiotic prescriptions (90.7%, 1763/1943) according to PPS from 2017 to 2019.

**Fig. 1:**
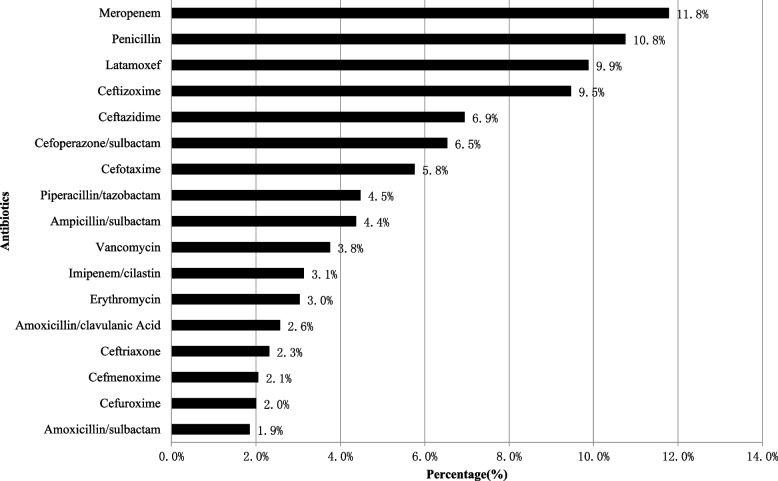
Antibiotics prescribing for neonates in China by drug utilization 90% in 2017–2019 (%)

In this survey, the five most commonly prescribed antibiotic agents for Chinese neonates include, meropenem (11.8%, 229/1943), penicillin (10.8%, 209/1943), latamoxef (9.9%, 192/1943), ceftizoxime (9.5%, 184/1943), and ceftazidime (6.9%, 135/1943), collectively accounting for 48.8% of all antibiotic prescriptions. The antibiotic distribution is shown in Fig. 1.

The five most common classes of antibiotics prescribed to neonates based on the Anatomical Therapeutic Chemical Classification (ATC) were third-generation cephalosporins (43.2%, 839/1943), carbapenems (15.1%, 293/1943), penicillin plus lactamase inhibitors (270/1943, 13.9%), penicillin (255/1943, 13.1%) and glycopeptides (94/1943, 4.8%).

### Antibiotic classes prescribed pattern based on the WHO access/watch/reserve group (WHO AWaRe)

46 types of antibiotics were included, based on the WHO AWaRe classification. There were 14 (30.4%) antibiotic agents in the Access group, accounting for 21.6% (419/1943) of the antibiotic prescriptions. There were 26 (56.5%) antibiotic agents in the Watch group, accounting for 69.1% (1343/1943) of the antibiotic prescriptions. There were two (4.3%) antibiotic agents in the reserve group, accounting for 0.3% (6/1943) of the antibiotic prescriptions. There were three (6.5%) antibiotic agents in the Not-recommended group, accounting for 9.0% (174/1943) of the antibiotic prescriptions. There was one (2.2%) antibiotic agent in the Unclassified group, accounting for 0.1% (1/1943) of the antibiotic prescriptions. The detailed antibiotic types in each group, based on the WHO AWaRe classification, are shown in Table 3.

**Table 3:** Antibiotics prescriptions for Chinese neonates based on WHO AWaRe classification

WHO AWaRe classification	Antibiotic agents	Frequency	Percentage
Access(*n* = 419)			
	Penicillin	209	49.9%
	Ampicillin/sulbactam	85	20.3%
	Amoxicillin/clavulanic acid	50	11.9%
	Metronidazole	24	5.7%
	Ampicillin	13	3.1%
	Cefazolin	11	2.6%
	Flucloxacillin	9	2.1%
	Amoxicillin	6	1.4%
	Amikacin	3	0.7%
	Cefathiamidine	3	0.7%
	Cloxacillin	2	0.5%
	Ornidazole	2	0.5%
	Oxacillin	1	0.2%
	Chloramphenicol	1	0.2%
Watch(*n* = 1343)			
	Meropenem	229	17.1%
	Latamoxef	192	14.3%
	Ceftizoxime	184	13.7%
	Ceftazidime	135	10.1%
	Cefotaxime	112	8.3%
	Piperacillin/tazobactam	87	6.5%
	Vancomycin	73	5.4%
	Imipenem/cilastin	61	4.5%
	Erythromycin	59	4.4%
	Ceftriaxone	45	3.4%
	Cefmenoxime	40	3.0%
	Cefuroxime	39	2.9%
	Cefepime	25	1.9%
	Teicoplanin	21	1.6%
	Piperacillin	14	1.0%
	Azithromycin	10	0.7%
	Cefotiam	3	0.2%
	Panipenem/betamipron	2	0.1%
	Ticarcillin	2	0.1%
	Cefaclor	2	0.1%
	Cefoperazone	2	0.1%
	Levofloxacin	2	0.1%
	Ertapenem	1	0.1%
	Mezlocillin	1	0.1%
	Cefdinir	1	0.1%
	Cefixime	1	0.1%
Reserve(*n* = 6)			
	Linezolid	4	66.7%
	Aztreonam	2	33.3%
Not recommended(*n* = 174)			
	Cefoperazone/sulbactam	127	73.0%
	Amoxicillin/sulbactam	36	20.7%
	Mezlocillin/sulbactam	11	6.3%
Unclassified(*n* = 1)			
	Ticarcillin/enzyme inhibitor	1	100%

### Antibiotic classes prescribed pattern based on the management of antibiotic classification (MAC) in China

According to the MAC in China, there were 413 (21.3%), 1090 (56.1%), 414 (21.3%), and 26 (1.3%) antibiotic prescriptions in the Unrestricted, Restricted, Special, and Unclassified groups, respectively.

47 antibiotic agents were included in this survey, of which 26 (55.3%) accounted for 90% of the prescriptions. There were 10 (21.3%) types of antibiotics in the Unrestricted group, accounting for 21.3% of antibiotic prescriptions. There were 20 (42.6%) types of antibiotic agents in the Restricted group, accounting for 56.1% of the antibiotic prescriptions. There were 7 (14.9%) types of antibiotic agents in the Special group, accounting for 21.3% of the antibiotic prescriptions. In the Special group, meropenem (55.3%) was the most common antibiotic, followed by vancomycin (17.6%). The top 5 prescribed antibiotics in the Restricted group were included into the third-generation cephalosporins. The detailed antibiotic types in each group, based on the antibiotic classification in China, are shown in Table 4.

**Table 4:** Antibiotics prescriptions for Chinese neonates bases on China classification

China classification	Antibiotic agents	Frequency	Percentage
Unrestricted(*n* = 413)			
	Penicillin	209	50.6%
	Erythromycin	59	14.3%
	Ceftriaxone	45	10.9%
	Cefuroxime	39	9.4%
	Metronidazole	24	5.8%
	Ampicillin	13	3.1%
	Cefazolin	11	2.7%
	Amoxicillin	6	1.5%
	Azithromycin(Oral)	5	1.2%
	Cefaclor	2	0.5%
Restricted(*n* = 1090)			
	Latamoxef	192	17.6%
	Ceftizoxime	184	16.9%
	Ceftazidime	135	12.4%
	Cefoperazone/sulbactam	127	11.7%
	Cefotaxime	112	10.3%
	Piperacillin/tazobactam	87	8.0%
	Ampicillin/sulbactam	85	7.8%
	Amoxicillin/clavulanic Acid	50	4.6%
	Cefmenoxime	40	3.7%
	Amoxicillin/ sulbactam	36	3.3%
	Piperacillin	14	1.3%
	Mezlocillin/sulbactam	11	1.0%
	Azithromycin(Intravenous)	5	0.5%
	Cefathiamidine	3	0.3%
	Cefotiam	3	0.3%
	Cefoperazone	2	0.2%
	Chloramphenicol	1	0.1%
	Mezlocillin	1	0.1%
	Cefdinir	1	0.1%
	Cefixime	1	0.1%
Special(*n* = 414)			
	Meropenem	229	55.3%
	Vancomycin	73	17.6%
	Imipenem/cilastin	61	14.7%
	Cefepime	25	6.0%
	Teicoplanin	21	5.1%
	Linezolid	4	1.0%
	Ertapenem	1	0.2%
Unclassified(*n* = 26)			
	Flucloxacillin	9	34.6%
	Amikacin	3	11.5%
	Aztreonam	2	7.7%
	Ornidazole	2	7.7%
	Cloxacillin	2	7.7%
	Panipenem/betamipron	2	7.7%
	Ticarcillin	2	7.7%
	Levofloxacin	2	7.7%
	Oxacillin	1	3.8%
	Ticarcillin/enzyme inhibitor	1	3.8%

### The antibiotic prescriptions pattern for acute pneumonia in Chinese children

Pneumonia was a predominant indicator of antibiotic prescriptions among neonates in China, with 858 prescriptions representing 44.2% of the total antibiotic prescriptions for neonates. The top five antibiotic agents, making up 50.7% of total antibiotic agents, were latamoxef (14.5%), ceftizoxime (11.4%), penicillin (10.1%), meropenem (7.3%), and cefoperazone/sulbactam (7.3%). The five main classes were third-generation cephalosporins (44.6%), beta-lactamase inhibitors (11.3%), penicillin (12.5%), carbapenems (11.2%), and macrolides (5.8%).

Per the WHO AWaRe classification, the Watch group antibiotics accounted for 69.6% (597/858), the Access group accounted for 22.1% (190/858), and the Not-recommended group accounted for 8.3% (71/858). No antibiotic in the Reserve group was for neonatal pneumonia in this study. According to the MAC in China, 180 (21.0%), 534 (62.2%) and 138 (16.1%) prescriptions were included in the Unrestricted, Restricted and Special groups respectively. In the Special group, meropenem (45.7%) and vancomycin (16.7%) were the predominant two antibiotics.

### The antibiotic prescriptions pattern for sepsis in Chinese neonates

In this study, 275 antibiotics were prescribed for the treatment of sepsis in neonates. The predominant antibiotics used were meropenem (25.8%), penicillin (11.3%), cefotaxime (10.5%), vancomycin (9.8%), and ampicillin/sulbactam (6.2%). The third-generation cephalosporins constituted 30.5% of total prescriptions for neonates with sepsis, followed by carbapenems (28.7%), penicillin (13.8%), glycopeptides (11.6%) and beta lactam-beta lactamase inhibitor (11.3%).

According to the WHO AWaRe classification, 69.5% (191/275) of prescribed antibiotics were in the Watch group, compared to 21.1% (58/275), 8.4% (23/275), and 1.1% (3/275), Access, Reserve and Unclassified groups respectively. According to the MAC in China, 46 (16.7%), 108 (39.3%), and 117 (42.5%) prescriptions were classified into the Unrestricted, Restricted and Special groups respectively. In the Special group, meropenem (60.7%) and vancomycin (23.1%) were the two most common antibiotics.

### The antibiotic prescriptions pattern for meningitis in Chinese neonates

90 antibiotic prescriptions were for meningitis. The three predominant antibiotics used were meropenem (33.3%), penicillin (18.9%) and ceftrixone (12.2%). The three main classes for meningitis were carbapenems (33.3%), third-generation cephalosporins (24.4%) and penicillins (22.2%).

Based on the WHO AWaRe classification, 63.3% of antibiotic prescriptions were in the Watch group, 30.0% in Access, 2.2% in Reserve. According to the MAC in China, 32 (35.6%), 18 (20.0%) and 39 (43.3%) prescriptions were included in the Unrestricted, Restricted and Special groups respectively.

## Discussion

Antimicrobial resistance poses a serious threat to children in China. A survey on bacterial epidemiology and antimicrobial resistance in Chinese children conducted in 10 children’s hospitals from 2016 to 2020 showed that carbapenem-resistant *Enterobacteriaceae* (CRE), carbapenem-resistant *P. aeruginosa* (CRPA), and carbapenem-resistant *A. baumannii* (CRAB) constituted 11.1, 20.1, and 26.8% of cases, respectively. CRE and CRPA are more common in neonates than in non-neonatal group [8]. Carbapenems are virtually the last resort for newborns, and highly carbapenem-resistant organisms (CROs) in neonates are commonly associated with high mortality rates. The broad-spectrum antibiotics may lead to higher potential antimicrobial resistance. The WHO AWaRe system and China’s antimicrobial classification are for antibacterial agents. The focus of this study was to show the pattern of antibacterial drugs for Chinese neonates.

In this survey, the two most common classes were third-generation cephalosporins (43.2%) and carbapenems (15.1%). This accounts for up to 58.3% in total which is far higher than other countries. Third-generation cephalosporins can easily promote bacterial resistance to β-lactam antibacterial drugs, and the overuse of carbapenems increases CROs, which may also result in over represented bacteria producing extra-spectrum β-lactamase (ESBL) and carbapenem resistance.

As described from a survey covering 56 countries, the top three antibiotics prescribed for neonates were gentamicin (28.8%), ampicillin (16.5%) and meropenem (12.7%) in Africa, ampicillin (29.1%), gentamicin (22.0%) and vancomycin (6.5%) in Americas, ampicillin (23.7%), cefotaxime (14.4%) and gentamicin (11.3%) in Eastern Mediterranean, gentamicin (19.6%), ampicillin (16.8%) and Benzylpenicillin (9.3%) in Europe, and ampicillin (17.3%), gentamicin (15.7%) and amikacin (14.2%) in South-East Asia [12]. In contrast, the top three antibiotics prescribed to neonates were meropenem (11.8%), penicillin G (10.8%), and latamoxef (9.9%) in China. One obvious difference from other countries is that gentamicin belonging to the Access group antibiotics, which was the first-line antibiotic in the Africa, Americas and Europe, is not considered for Chinese neonates. Only three aminoglycoside prescriptions (amikacin) of the total (1943) antibiotic prescriptions were found in this survey. This may be related to antimicrobial management policies in China. Gentamicin was prohibited or requested to monitor the concentration in children younger than 6 years of age in China, while very few hospitals were able to monitor the concentration [13]. The ototoxicity of aminoglycoside antibiotics is the main barrier to be prescribed for children in China. The ototoxicity of gentamicin is genetic susceptible, but the carrier rate of *A1555G* in Chinese newborns was lower than in European children [14, 15]. To date, there is no evidence that Chinese children are more susceptible to gentamicin-induced deafness than children from other countries. At the same time, the non-use of aminoglycosides has led to the overuse of third-generation cephalosporins and carbapenems.

In Chinese neonates, the resistance rates of gentamicin and amikacin to common pathogens were very low, much lower than third-generation cephalosporins and carbapenems. A study of the epidemiology and drug resistance of neonatal bloodstream infections in a medical center in East China from 2016 to 2020 reported that the resistance rates of *Escherichia coli* and *Klebsiella pneumonia* to amikacin were 0 and 1.96%, respectively. The resistance rate of *K. pneumonia* to gentamicin was 7.14%, 69.23 to cefotaxime and 32.00% to meropenem [16]. Gentamicin is inexpensive and effective in Chinese neonates. In the future, genetic screening before the use of aminoglycosides will enhance the confidence of Chinese doctors and patients regarding the use of aminoglycosides.

Bacterial resistance is closely associated with broad-spectrum antibiotic use. The WHO AWaRe classification is a simple and efficient tool for evaluating antibiotic applications [9, 10]. As for the WHO AWaRe classification, 69.1% of antibiotic prescriptions were in the Watch group and only 21.6% were in the Access group, indicating that the proportion of access antibiotics usage was far lower than the goal of the WHO (60%). In this survey, four of the top five antibiotic agents prescribed to Chinese neonates (38.1%) are included in the Watch group. The proportion of Watch group antibiotics in china was the second highest [12]. According to the MAC in China, 21.3% of antibiotic prescriptions were included in the Special group antibiotics which are restricted strictly in China. For pneumonia which is a kind of non-invasive infectious disease, the Watch group and Special group accounted for 69.6 and 16.1%. The antibiotics in the Watch group and the Special group have a higher resistance potential, are more expensive and have more side-effects.

In the Watch group, the cephalosporins and macrolides were mixed with carbapenems and glycopeptides, which are more expensive and have more side effects, they are usually used as a last resort in children. Nevertheless, the Special group (Chinese Antibiotic Classification) included antibiotics like meropenem, linezolid, and vancomycin and excludes second cephalosporins, third cephalosporins, and macrolides. WHO AWaRe and the MAC in China together will be a facilitative and complementary metric tool for the categorization of antibiotics.

In this survey, pneumonia was the predominant indication for antibiotics in Chinese neonates, accounting for 44.2% of all prescriptions. The third-generation cephalosporins (44.6%) and carbapenems (11.2%) in Chinese neonates accounted for 55.8%, but these third-generation cephalosporins and carbapenems were unnecessary. A single-center study on pathogen and antimicrobial resistance in Chinese neonates with community-acquired pneumonia or hospital-acquired pneumonia illustrated that the top three types of bacteria were *Klebsiella pneumonia, Escherichia coli* and *Staphylococcus aureus*, with 26.9% of gram-negative bacteria producing extra-spectrumβ-lactamase (ESBL). Penicillin plus lactamase inhibitors should be the first-line treatment for neonates with pneumonia [17]. A skin test is required before penicillin or penicillins plus β-lactamase inhibitor, whether oral or intravenous. This is the biggest obstacle for prescribepenicillins. As an alternative, the third-generation cephalosporins are commonly chosen. Meropenem accounted for 7.30% for neonatal pneumonia and 25.8% for sepsis. There were several reasons why meropenem was used so frequently. Gentamicin was prohibited in newborns in China due to possible ototoxicity, thus meropenem was chosen as an alternative for gram-negative bacteria infections. The proportion of ESBL-producing *Escherichia coli.*(27.2%) and *Klebsiella pneumonia*(55.7%)were high in Chinese neonates [16]. Meropenem was overused in China. Antibiotics like meropenem can be initially used for patients with sepsis because of the high proportion of - *Enterobacterales* producing ESBLs, but the use of antibiotics should be downgraded according to the antimicrobial susceptibility results when the pathogen is identified. Moreover, piperacillin tazobactam can be chosen as an alternative for *Enterobacterales* producing ESBLs.

This study has some limitations. Information on the distribution of pathogens and antimicrobial resistance from cooperative medical institutions was lacking, making it difficult to evaluate the rationality of antibiotic prescriptions. In future studies, information on the pathogens and antimicrobial resistance should be collected simultaneously. Moreover, as this was a cross-sectional study, data on the duration of antibiotic use and patient outcomes could not be collected. Furthermore, the voluntary nature of this study and the varying amounts of data uploaded by different hospitals may have influenced the representativeness of the results.

The strengths of this study lie in the fact that the collaboration of 15 hospitals from nine provinces provided the largest dataset of neonatal antibiotic prescriptions in China. In addition, this study is the first to describe the pattern of antibiotic prescription in Chinese neonates by combining the WHO AWaRe classification and China’s administrative classification. Moreover, the simplicity and high feasibility of the point-prevalence survey, it may be an effective method for the continuous monitoring of antibiotic use.

### Supplementary Information

**Additional file 1: Supplementary Table 1.** Antibiotics prescribed based on WHO AWaRe and Antibiotic Classification in China.

## Data Availability

The raw data supporting the conclusions of this article are available from the corresponding author (Dr. Yonghong Yang) upon reasonable request.
