# Supplementary material for: Patterns of antibiotic administration in Chinese neonates: results from a multi-center, point prevalence survey
Source: BMC Infect Dis. 2024 Feb 12;24:186. doi: 10.1186/s12879-024-09077-7 (PMC10863225; doi:10.1186/s12879-024-09077-7)
Supplement: Supplementary file 1 — Additional file 1: Supplementary Table 1. Antibiotics prescribed based on WHO AWaRe and Antibiotic Classification in China. [file 12879_2024_9077_MOESM1_ESM.docx]

Supplementary table 1 Antibiotics prescribed based on WHO AWaRe and Antibiotic Classification in China

| Antibiotic | WHO AWaRe | Antibiotic classification in China |
| --- | --- | --- |
| Amikacin | Access | Unclassified |
| Amoxicillin | Access | Unrestricted |
| Amoxicillin/clavulanic Acid | Access | Restricted |
| Amoxicillin/sulbactam | not recommended | Restricted |
| Ampicillin | Access | Unrestricted |
| Ampicillin and enzyme inhibitor | Access | Restricted |
| Azidocillin | Access | Unclassified |
| Azithromycin(IV) | Watch | Restricted |
| Azithromycin(Oral) | Watch | Unrestricted |
| Azlocillin | Watch | Unclassified |
| Aztreonam | Reserve | Unclassified |
| Benzylpenicillin | Access | Unrestricted |
| Cefaclor | Watch | Unrestricted |
| Cefaloridine | Access | Unclassified |
| Cefamandole | Watch | Unclassified |
| Cefatrizine | Access | Restricted |
| Cefazedone | Access | Unclassified |
| Cefazolin | Access | Unrestricted |
| Cefbuperazone | Watch | Unclassified |
| Cefdinir | Watch | Restricted |
| Cefepime | Watch | Special |
| Cefixime | Watch | Restricted |
| Cefmenoxime | Watch | Restricted |
| Cefmetazole | Watch | Unclassified |
| Cefminox | Watch | Unclassified |
| Cefodizime | Watch | Restricted |
| Cefoperazone | Watch | Restricted |
| Cefoperazone Sulbactam | not recommended | Restricted |
| Cefotaxime | Watch | Restricted |
| Cefotiam | Watch | Restricted |
| Cefoxitin | Watch | Unclassified |
| Cefpodoxime | Watch | Restricted |
| Cefradine | Access | Unclassified |
| Ceftazidime | Watch | Restricted |
| Ceftezole | Access | Unclassified |
| Ceftizoxime | Watch | Restricted |
| Ceftriaxone | Watch | Unrestricted |
| Cefuroxime | Watch | Unrestricted |
| Ciprofloxacin | Watch | Unclassified |
| Clarithromycin | Watch | Unrestricted |
| Clindamycin | Access | Unrestricted |
| Ertapenem | Watch | Special |
| Erythromycin | Watch | Unrestricted |
| Fosfomycin | Reserve | Unrestricted |
| Fusidic acid | Watch | Restricted |
| Imipenem/cilastin | Watch | Special |
| Latamoxef | Watch | Restricted |
| Levofloxacin | Watch | Unclassified |
| Linezolid | Reserve | Special |
| Meropenem | Watch | Special |
| Metronidazole | Access | Unrestricted |
| Mezlocillin | Watch | Restricted |
| Mezlocillin and sulbactam | not recommended | Restricted |
| Nitrofurantoin | Access | Unrestricted |
| Ofloxacin | Watch | Unclassified |
| Ornidazole | Access | Unclassified |
| Oxacillin | Access | Unclassified |
| Panipenem and betamipron | Watch | Unclassified |
| Piperacillin | Watch | Restricted |
| Piperacillin and enzyme inhibitor | Watch | Restricted |
| Roxithromycin | Watch | Unrestricted |
| Sulbenicillin | Watch | Unclassified |
| Sulfamethoxazole and trimethoprim | Access | Unrestricted |
| Ticarcillin and enzyme inhibitor | Unclassified | Unclassified |
| Tigecycline | Reserve | Special |
| Tobramycin | Watch | Unclassified |
| Vancomycin | Watch | Special |
